# Diseases of Pre-Historic Man

**Published:** 1883-10

**Authors:** 


### DISEASES OF PRE-HISTORIC MAN.

This important part of archæological anthropology has received the attention of a number of competent investigators, and the subject is growing in importance with the advance in archæological knowledge, as a means of tracing the etiology of various maladies. At present, for instance, the medical world is in doubt as to the source of that dread scourge, syphilis, and further research is needed to definitely determine whether it be of ancient or modern origin. No positive evidence of its existence among pre-historic men has yet been produced, although a number of investigators claim to have found it. A syphilitic skull was lately found by the editor of this journal at the Peabody Museum of Archæology, at Cambridge, but an inquiry into its history developed the fact that it was quite possibly of recent origin, and not the remains of a pre-historic man.

According to *Gaillard's Medical Journal*, an important paper was lately read by M. Le Baron, before the Anthropological Society of Paris. Dr. Knapp, of New York, has also made a study of the mound-builders from an otological standpoint. As a result of the examination of two hundred and fifty skulls he found exostoses in forty-four. He ascribes this to the habit of carrying foreign bodies in the meatus auditorius. Mr. W. H. Jackson, M. R. C. S., of England, in a paper read before the Medico-Chirurgical Society of West Kent, cites a case of exostosis of the femur found at Lozere; evidences of toothache and abscesses of the jaw, of rheumatic ulceration of the joint in a jaw-bone, and an astragulus, found in Belgian bone caves; cases of hydrocephalus, hemiplegia, hip-joint disease, and of synostosis, or bony union of the sutures in the celebrated Neanderthal skull, the oldest one known, and many other traces of pre-historic disease and abnormality.

So far as we are aware the diseases of the teeth have not attracted the study which their relative importance as a means of determining the condition of the early races would seem to demand. To the dental anatomist these organs are an open book, upon whose pages may be read very much of the life history of the extinct races that formerly inhabited this continent. Recent investigation has determined that many of the eruptive diseases leave indelible traces upon the teeth, more especially during their formative period, and a yet closer study of the histology of the dental organs of pre-historic man will undoubtedly reward the observer with many hitherto unknown facts concerning this people.

Much may be learned, too, of the habits of a race by an examination of their teeth. If their food was of a hard, gritty character, the teeth will show extensive wear. The south-western mound-builders undoubtedly lived upon hard grains, like maize, for their teeth were very much abraded. The ancient Peruvians subsisted upon a food which permitted extensive accumulations of tartar, or salivary calculus, while the Sandwich Islanders lived largely upon succulent fruits, which contained sufficient acid to prevent any such encrustations. The north-western Indians probably existed mainly by the chase, and the condition of their teeth plainly shows that theirs was largely an animal diet.

In another department of this number of the INDEPENDENT PRACTITIONER, we give place to the record of some observations made upon pre-historic skulls by the Editor, which would have been manifestly out of place here. They are not of such an elaborate character as to demand any particular scientific attention, but they indicate that the theories held by many intelligent dentists need careful revision, and they open a field for more minute investigation. As soon as the exigencies of a busy life permit, we propose to try and study the subject yet further.

In the Peabody Museum are many interesting specimens which indicate a rude but effective kind of surgery among early American races, as well as remarkable tolerance of foreign substances, and recovery from what would have seemed to be mortal wounds. In one case both plates of one of the parietal bones had been cut through by some incisive weapon, probably a stone hatchet, making a wound of a couple of inches in length, but the fragments had evidently been carefully removed, the injured plates raised, and the wound so dressed that there had been complete or partial recovery, and the man had evidently lived for a number of years after the injury.

In another case a flint arrow-head had penetrated at the inner canthus of one of the eyes and there been broken off, leaving about three-fourths of an inch of it among the ethmoidal cells, yet the wound had healed and the opening through the orbital bone had nearly or quite closed.

